# Feasibility and Acceptability of Chatbots for Nutrition and Physical Activity Health Promotion Among Adolescents: Systematic Scoping Review With Adolescent Consultation

**DOI:** 10.2196/43227

**Published:** 2023-05-05

**Authors:** Rui Han, Allyson Todd, Sara Wardak, Stephanie R Partridge, Rebecca Raeside

**Affiliations:** 1 Engagement and Co-Design Research Hub School of Health Sciences, Faculty of Medicine and Health University of Sydney Westmead Australia

**Keywords:** chatbot, artificial intelligence, text message, adolescent nutrition, physical activity, health promotion

## Abstract

**Background:**

Reducing lifestyle risk behaviors among adolescents depends on access to age-appropriate health promotion information. Chatbots—computer programs designed to simulate conversations with human users—have the potential to deliver health information to adolescents to improve their lifestyle behaviors and support behavior change, but research on the feasibility and acceptability of chatbots in the adolescent population is unknown.

**Objective:**

This systematic scoping review aims to evaluate the feasibility and acceptability of chatbots in nutrition and physical activity interventions among adolescents. A secondary aim is to consult adolescents to identify features of chatbots that are acceptable and feasible.

**Methods:**

We searched 6 electronic databases from March to April 2022 (MEDLINE, Embase, Joanna Briggs Institute, the Cumulative Index to Nursing and Allied Health, the Association for Computing Machinery library, and the IT database Institute of Electrical and Electronics Engineers). Peer-reviewed studies were included that were conducted in the adolescent population (10-19 years old) without any chronic disease, except obesity or type 2 diabetes, and assessed chatbots used nutrition or physical activity interventions or both that encouraged individuals to meet dietary or physical activity guidelines and support positive behavior change. Studies were screened by 2 independent reviewers, with any queries resolved by a third reviewer. Data were extracted into tables and collated in a narrative summary. Gray literature searches were also undertaken. Results of the scoping review were presented to a diverse youth advisory group (N=16, 13-18 years old) to gain insights into this topic beyond what is published in the literature.

**Results:**

The search identified 5558 papers, with 5 (0.1%) studies describing 5 chatbots meeting the inclusion criteria. The 5 chatbots were supported by mobile apps using a combination of the following features: personalized feedback, conversational agents, gamification, and monitoring of behavior change. Of the 5 studies, 2 (40.0%) studies focused on nutrition, 2 (40.0%) studies focused on physical activity, and 1 (20.0%) focused on both nutrition and physical activity. Feasibility and acceptability varied across the 5 studies, with usage rates above 50% in 3 (60.0%) studies. In addition, 3 (60.0%) studies reported health-related outcomes, with only 1 (20.0%) study showing promising effects of the intervention. Adolescents presented novel concerns around the use of chatbots in nutrition and physical activity interventions, including ethical concerns and the use of false or misleading information.

**Conclusions:**

Limited research is available on chatbots in adolescent nutrition and physical activity interventions, finding insufficient evidence on the acceptability and feasibility of chatbots in the adolescent population. Similarly, adolescent consultation identified issues in the design features that have not been mentioned in the published literature. Therefore, chatbot codesign with adolescents may help ensure that such technology is feasible and acceptable to an adolescent population.

## Introduction

Adolescents, aged 10-19 years, as defined by the World Health Organization (WHO), are a unique age group, who begin to develop independent lifestyle habits that they carry into adulthood [1]. Concerningly, the prevalence of overweight and obesity among adolescents is increasing worldwide. In 2016, more than 31 million children and adolescents aged 5-19 years were reported as overweight or obese [1]. Overweight and obesity in adolescence are associated with poorer health outcomes in adulthood, including cardiovascular disease and type 2 diabetes [2]. Therefore, intervening early in the life course is critical to prevent the future burden of chronic disease and comorbidities [3,4]. Regular physical activity and optimal nutrition are fundamental in preventing and assisting those with overweight and obesity to return to a healthy weight. Worldwide, more than 80% of adolescents do not meet the recommended levels of physical activity or sedentary behavior guidelines [4]. Since the COVID-19 pandemic began, research has reported increased screen time being associated with weight gain among adolescents [2]. Additionally, most adolescents fail to meet WHO’s guidelines on daily fruit and vegetable intake [5]. The overconsumption of nutrient-poor, ultraprocessed foods and sugar-sweetened beverages is further contributing to the rising rates of overweight and obesity. Simultaneously, malnutrition, micronutrient deficiencies, and food insecurity continue to persist among adolescents worldwide [6]. Adolescents need support to improve physical activity and nutrition behaviors, which in turn will minimize the growing rate of adolescents with overweight and obesity worldwide.

Digital health interventions, such as mobile apps, text messaging, and gamification, show promise for improving the health of adolescents through targeting physical activity and dietary behaviors [7,8]. Nearly 70% of adolescents in high-income countries have a smartphone and are frequently online [9]. Mobile-based interventions are relatively low cost, accessible, and widely acceptable among adolescents [10]. Gamification is the implementation of game design elements in real-world contexts for nongaming purposes [11] and has been found to be effective in improving physical activity levels, fruit and vegetable intake, and nutrition knowledge in adolescents [12,13]. For example, the popular online game Pokémon Go has been found to promote physical activity [7,14]. Mobile apps may assist in improving adolescents’ health with a plethora of apps available. A review by Schoeppe et al [15] found that currently available mobile apps that promote physical activity and nutrition have moderate quality and use a range of behavior change techniques, such as encouragement, performance feedback, and gamification. However, there is limited knowledge of user engagement [15]. A randomized controlled trial, conducted in 14 secondary schools in Australia, evaluated the influence of a mobile app to promote physical activity in adolescents and found that half of the participants were influenced by the “push-prompt” message reminder to be active, reduce sweetened beverage consumption, and reduce screen time [16]. Further, the use of semipersonalized text messaging has been found to be a feasible and acceptable strategy to engage adolescents to promote healthy behaviors [17]. Incorporating gamification and personalized feedback may help improve engagement for young people in digital health interventions [13]. As technology continues to evolve, it is important to evaluate emerging features to help improve and sustain diet and physical activity behaviors among adolescents [7,18].

Artificial intelligence (AI) is a rapidly developing technical science being applied to the health care field [8,19]. It is commonly used in precision medicine, using machine learning, which involves training models with data [19]. The use of natural language processing (NLP) allows AI to communicate using humanlike language, as well as to extract and construct information from social media and medical documents [8]. AI items, such as Apple Siri and Google Assistant, are becoming increasingly popular among the public to answer health-related questions [20,21]. Chatbots are an emerging software application designed for text-based conversation. They can search for information from the internet or a database to respond to users’ inquiries and personalize communication with humans [22]. Chatbots can be designed with or without AI. Those without AI cannot learn and adapt and often have predetermined responses based on the question asked by the user. However, AI chatbots are trained to have humanlike conversations using NLP. Therefore, there is potential for the use of chatbots as a digital health intervention to improve nutrition and physical activity behaviors across the life course. There is current evidence of chatbots promoting physical activity in the adult population, which is encouraging, but further research is needed to support these findings [23]. A systematic review investigating the use of chatbots to improve physical activity and nutrition across all age groups found no studies specifically targeting adolescents [23]. Chew’s [24] recent scoping review of chatbots used to promote weight loss across all age groups also found the same gap in knowledge and highlighted the importance of using age-appropriate design features to enhance engagement for adolescents There is potential for this cost-effective and highly accessible technology to deliver health information to young people to improve their nutrition and physical activity behaviors [25]. However, there is limited research on the feasibility and acceptability of chatbots in the adolescent population [23]. This systematic scoping review aims to evaluate findings from peer-reviewed, published studies to understand the feasibility and acceptability of chatbots to promote nutrition and physical activity in adolescents. A secondary aim is to identify design features of chatbots that would be acceptable and feasible with an established youth advisory group.

## Methods

### Study Design

A scoping review was determined to be the most suitable method to synthesize data to identify knowledge gaps and look broadly at the existing literature [26]. The systematic scoping review methodology was informed by the 6-stage methodological framework outlined by Arksey and O’Malley [27] and the Joanna Briggs Institute guidelines for scoping reviews [28]. The review was reported following the Preferred Reporting Items for Systematic Reviews and Meta-Analyses extension for scoping reviews (PRISMA-ScR) [29]. The research questions were formulated by the research team, along with the eligibility criteria for including relevant studies. Next, studies were selected based on the predefined eligibility criteria, and relevant data from the included studies were extracted. Following data extraction, results were collated and summarized narratively.

### Eligibility Criteria

To be included, peer-reviewed research studies must have (1) been conducted in the adolescent population, defined according to WHO as the second decade of life (10-19 years); (2) participants without a chronic disease, except obesity or type 2 diabetes; (3) assessed the feasibility and acceptability of chatbots used for nutrition or physical activity interventions or both that encourage individuals to meet dietary or physical activity guidelines and support positive behavior change; (4) been conducted in 2010 and beyond (to coincide with the period that smart devices were normalized in society, including chatbots); and (5) been written in any language and conducted in any country. Quantitative and qualitative peer-reviewed papers were included. For this study, chatbots were defined as programs that contained a conversational agent that could engage in “small talk”; smart conversational agents, such as Apple Siri; and those involving a computer-generated virtual agent.

### Search Strategy

Initially, a limited search of Google and MEDLINE was completed by the authors to evaluate the scope of existing research in the literature. The search strategy was developed in conjunction with the academic liaison librarian. An advanced search was conducted in March 2022 using MEDLINE, including Medical Subject Headings (MeSH) and keyword searches, in 3 core concept areas: chatbots, nutrition intervention, and physical activity intervention. An extensive list of synonyms for all terms was included to capture the maximum number of studies (Multimedia Appendix 1). Once key concepts and terms were determined, the search strategy was adapted to other database searches. The search was implemented using 6 electronic databases (MEDLINE, Embase, Joanna Briggs Institute [JBI], the Cumulative Index to Nursing and Allied Health [CINAHL], the Association for Computing Machinery [ACM] library, and the IT database Institute of Electrical and Electronics Engineers [IEEE]). We also conducted gray literature searches to identify any papers that may have been missed through the search.

### Screening and Study Selection

All search results were stored in an Endnote library (Endnote X9.3.3, Clarivate), and duplicates were removed. Next, the Endnote library was uploaded to Covidence (Veritas Health Innovation Ltd), and additional duplicates were removed. The PRISMA-ScR model was used to screen and select studies. Title and abstract screening and full-text screening were conducted based on the inclusion criteria. Two reviewers (authors RH and SW) performed the source selection independently. Any disagreements were discussed between the 2 reviewers, and if the conflicts were not resolved, further discussion with a third reviewer (author RR) was undertaken.

### Data Exaction and Presentation of Results

Two authors conducted data extraction independently (authors RH and AT), with consensus provided by a third reviewer (RR). The data were extracted using predeveloped data extraction tables. The extracted results were descriptively mapped in tables and a narrative summary.

### Consultation Exercise

One author (RR) presented an overview of the results of the scoping review to an established youth advisory group, which includes 16 adolescents aged 13-18 years, residing in New South Wales, Australia (Health Advisory Panel for Youth at the University of Sydney [HAPYUS]). The youth advisory group was recruited via social media advertising and went through a competitive selection process. They serve a 12-month term on the panel, providing their input to several adolescent research projects [30,31]. The results were presented to the youth advisory group to gain valuable insights into issues relating to the results that the scoping review alone would not have alerted the research team to. After presentation of the scoping review, 2 members of the youth advisory group volunteered to lead a statement on behalf of the group, included in the Results section, relating to considerations for researchers or developers working in this area. This statement was written by HAPYUS in their own words.

### Ethical Considerations

Ethics approval was not required. The adolescents who took part in the consultation were considered members of our research team.

## Results

### Study Selection

**Figure 1:**
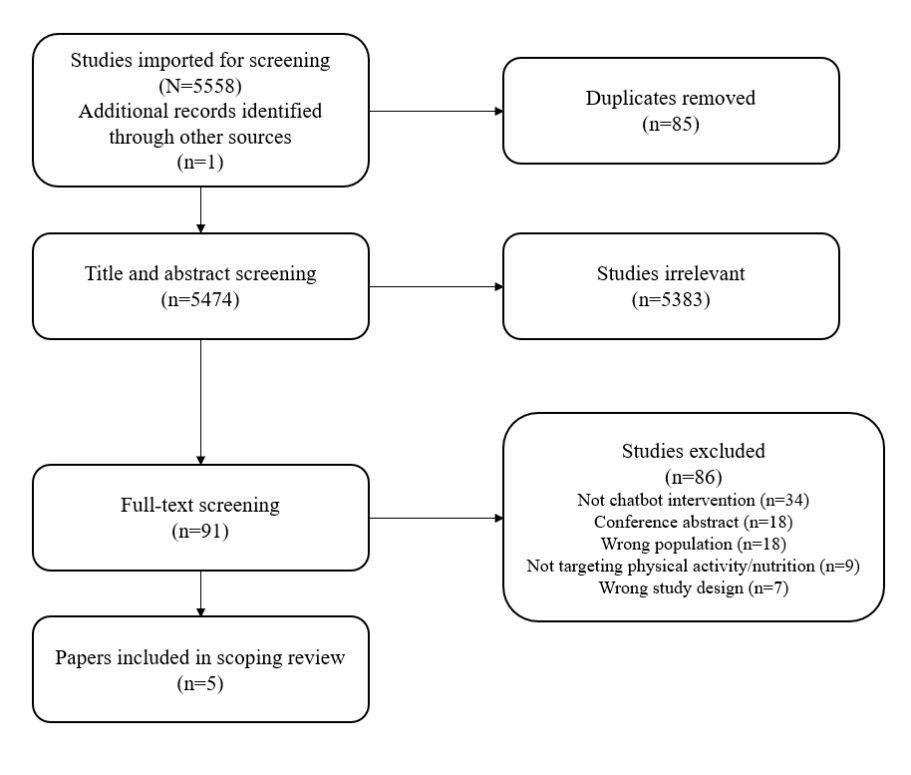
PRISMA-ScR flow diagram. PRISMA-ScR: Preferred Reporting Items for Systematic Reviews and Meta-Analyses extension for scoping reviews.

The search identified 5558 papers that were imported for screening, and 85 (1.5%) duplicates were removed. After title and abstract screening, 5383 (98.4%) of 5473 papers were excluded. The remaining 90 (1.6%) full-text papers were screened, and 86 (95.6%) papers were excluded. Overall, a total of 4 (4.4%) relevant papers were identified through database searching. One additional paper was discovered through gray literature searching (Figure 1). The 5 studies were conducted in different countries: Korea, India, Finland, Switzerland, and Belgium. Among the 5 studies, 3 (60.0%) interventional studies were identified, 2 (66.7%) of which were randomized controlled trials (RCTs) and 1 (33.3%) was a pre-post study. In addition, 1 (20.0%) of the 5 studies was an exploratory analysis as a subset of an RCT and 1 (20.0%) was a mixed methods pilot study. A narrative summary of the results of the included studies and characteristics of chatbots is presented in Tables 1 and 2, respectively.

**Table 1:** Key characteristics of included studies.

First author, year, country	Study design	Recruitment	Participants, N	Sex	Age range (years)	Aim	Use of codesign in chatbot development	Dropout
Lee, 2017, Korea [32]	Pre-post intervention	Students from 2 same-sex high schools in Seoul, Korea	33	Female: n=24, 72.7%; Male: n=9, 27.3%	16-18	To test the feasibility of a mobile app Diet-A and examine whether Diet-A could be used to monitor dietary intake among adolescents	N/A^a^	N/A
Padman, 2017, India [33]	Exploratory analysis	Students from 3 middle schools in urban India recruited for an RCT and deidentified participants from the RCT recruited in the explanatory analysis	14	Female: n=7, 50.0%; Male: n=7, 50.0%	10-11	To analyze game telemetry to understand user interactions from playing Fooya! and provide new insight for designing interventions via games to improve pediatric overweight and obesity rates	N/A	N/A
Pyky, 2017, Finland [34]	RCT^b^	Males who for conscripted for military service in Finland	496	Male: n=100, 100.0%	Mean 17.8	To assess whether a tailored mobile physical activity intervention can improve life satisfaction and self-rated health among young adolescent men	16-20-year-old males involved in the design, development, and testing of the mobile service	Lost to follow-up: n=151, 30.4%; Controls: n=167, 33.7%; Intervention: n=135, 27.2%
Stasinaki, 2021, Switzerland [35]	RCT	Children’s Hospital of Eastern Switzerl (specialized childhood obesity management center)	31	Female: n=13, 41.9%; Male: n=18, 58.1%	10-18	To assess whether PathMate2 can improve the BMI (kg/m^2^), physical capacities, and stress parameters in adolescents with obesity, under the supervision of pediatric obesity experts	N/A	Lost to follow-up: 0.1%
Maenhout, 2021, Belgium [36]	Mixed methods pilot study	Flemish secondary schools	Phase 1: 36; Phase 2: 6; Phase 3: 81	Phase 1: Female: n=29, 80.6%; Male: n=7, 19.4%; Phase 2: Female: n=6, 100.0%; Phase 3: N/A	12-15	To assess the feasibility and engagement of a chatbot protype among adolescents to promote healthy behaviors	Phase 1: focus groups to inform the development of the chatbot prototype, including content and design; Phase 2: pretest of the protype	Phase 3: quit after receiving a wrong answer from the chatbot: n=61, 66.7%

^a^N/A: not applicable.

^b^RCT: randomized controlled trial.

**Table 2:** Summary of chatbots.

First author, year, country	Chatbot name	Intervention delivery	Conversational agent	Gamification	Personalized feedback	Monitored behavior change
Lee, 2017, Korea [32]	Diet-A	Mobile app	Yes	No	Yes	Yes
Padman, 2017, India [33]	Fooya!	Mobile app	No	Yes	No	No
Pyky, 2017, Finland [34]	MOPOrtal	Mobile service	Yes	Yes	Yes	Yes
Stasinaki, 2021, Switzerland [35]	PathMate2	Mobile app	Yes	Yes	Yes	Yes
Maenhout, 2021, Belgium [36]	Self-regulation app	Mobile app	Yes	No	Yes	Yes

### Overview of Included Studies

Studies recruited adolescents aged 10-19 years. Of the 5 studies, 4 (80.0%) had small sample sizes with varying distributions of male and female participants. In addition, 1 (20.0%) study had an even distribution of males and females [33], 2 (40.0%) studies had more than 70.0% female participants [32,36], and 2 (40.0%) studies had predominantly (58.0% and 100.0%, respectively) male participants [34,35]. An overview of the included studies is provided in Table 1, including key characteristics of the included studies (eg, authors, year of publication, country, aim, study type, participant characteristics). In the included studies, 1 (20.0%) study included adolescents with overweight or obesity recruited from a hospital setting [35]. The other 4 (80.0%) studies included participants who were otherwise healthy [32-34,36]. In addition, 3 (60.0%) studies were conducted in school settings, of which 2 (66.7%) were conducted in high schools and 1 (33.3%) in middle schools [32,33,36]. Furthermore, 1 (20.0%) study recruited only males eligible for military conscription [34].

### Summary of Chatbots

The 5 chatbots were supported by mobile apps (n=4, 80.0%) or web applications delivered via mobile devices (n=1, 20.0%). The 5 chatbots were different in their delivery. The chatbots used a combination of 4 features, namely a conversational agent (n=4, 80.0%), gamification (n=3, 06.0%), personalized feedback (n=4, 80.0%), and monitoring of behavior change (n=4, 80.0%). An overall summary of the chatbots is provided in Table 2, including the characteristics of the intervention in more detail (chatbot details, intervention details, outcomes and key findings that relate to the scoping review question). In the 5 studies, chatbots were used in different ways to improve adolescents’ nutrition and physical activity behaviors. Nutrition was the focus of 2 (40.0%) studies, in which chatbots targeted nutrition intake and food choice [32,33]. Physical activity was the focus of 2 (40.0%) studies, in which chatbots targeted physical activity, physical capacity, and the BMI [34,35]. Finally, 1 (20.0%) study had a chatbot that targeted both nutrition and physical activity behaviors [36]. Each intervention targeted nutrition and physical activity differently. Diet-A used a mobile app where the participants recorded their dietary intake and provided real-time, personalized feedback on their diet [32]. Fooya! was an interactive mobile game and AI robot that aimed to influence healthy food choices [33]. The 2 chatbots targeting physical activity had unique features in their delivery to help participants achieve their goals. PathMate2 was a virtual health coach [35], and MOPOrtal was a web-based interface with a combined mixed-reality game [34]. The self-regulation app that targeted physical activity and nutrition behaviors allowed participants to ask the chatbot questions about physical activity, sedentary behavior, breakfast intake, and mental health [36].

### Summary of Feasibility and Acceptability of Chatbots

Overall, there were mixed reports of the feasibility and acceptability of chatbots across all 5 studies. Of the participants who used Diet-A, 61.9% (13/21) said they were satisfied with it to monitor their dietary intake, 65.0% (13/20) said it was helpful, and 57.1% (12/21) agreed that they were able to learn about their dietary intake. However, 71.4% (15/21) of the participants reported that it was burdensome and 85.7% (18/21) reported that they sometimes forgot to record their diet [32]. In the Fooya! mobile app, participants gained knowledge and awareness of healthy food, but engagement decreased throughout the game [33]. In the MOPOrtal intervention, there were low overall intervention effects, except in participants who reported poorer health at baseline. No other data on feasibility or acceptability were reported [34]. PathMate2 was still being used by just over half of the participants (51.0%) at 6 months. The average app usage rate was 71.5%, and the average adherence rate was 57.2% during the intervention [35]. Finally, for the self-regulation app, 74.1% (60/81) of participants used the chatbot during the pilot; however, two-thirds of these participants quit and did not ask any further questions if the chatbot gave a wrong answer [36].

### Summary of Health Outcomes

Of the 5 studies, 3 (60.0%) studies recorded and analyzed participants’ health-related characteristics at baseline and after the intervention, with the length of the interventions ranging from 3 to 6 months [32,34,35], and 1 (20.0%) study had an additional 6-month maintenance phase to measure sustained changes [35]. The Diet-A intervention used the CAN-Pro 4.0 program to assess nutrient intake through 24-hour recalls pre- and postintervention. This study found that participants had a significant reduction in sodium and calcium intake and an increase in fruit and vegetable consumption. However, there was no improvement in overall diet among the participants following the intervention [32]. MOPOrtal measured daily minutes of physical activity through a physical activity monitor and collected height and weight to calculate the BMI. It demonstrated a limited increase in physical activity and increased mean weight in both intervention and control groups. Only those men with low life satisfaction and poor self-rated health at baseline were associated with improved satisfaction postintervention [34]. Finally, PathMate2 measured the BMI-SDS (where SDS refers to the standard deviation score) and other anthropometric measures and found that participants can improve physical capacity, increase muscle mass, and reduce body fat percentage following use of the intervention, but there was no sustained significant change in the BMI-SDS [35]. The other 2 (40.0%) studies did not measure any health-related characteristics. A full summary of outcomes is provided in Multimedia Appendix 2.

### Summary of Chatbot Development

Of the 5 chatbots, 4 (80.0%) used text-based mobile apps yet were developed in different ways, including based on health databases, transtheoretical models, scientific evidence, and the person-based approach (PBA) [32,34-36]. The mobile app Diet-A, developed by Lee et al [32], is a self-monitoring app to help participants record their diet and offers real-time feedback and disease prevention information based on dietary reference intakes for Koreans. The feedback and disease prevention information were built under 3 health and food-related databases, and nutrient content information was provided by external stakeholders [32]. MOPOrtal can deliver tailored health information and feedback messages in line with Finnish national physical activity recommendations for 13-18-year-olds. The messages delivered were based on the transtheoretical model of behavior change. The given message was different at each intervention stage to match the process of change theorized and provide the most appropriate information to the participants. The health information was based on the reviewed scientific evidence [34]. The PathMate2 mobile app included a conversational agent as a virtual coach and was developed with MobileCoach open source software. This agent can chat with participants and encourage them to achieve the challenge of staying healthy through physical activity according to Swiss physical activity guidelines. PathMate2 aimed to support behavior change using goal setting, self-monitoring, stimulus control, and behavioral contracting to support a healthy lifestyle [35]. Finally, Maenhout et al [36] used PBA to ensure the needs and perspectives of the end user were embedded in the guiding principles of the chatbot, and therefore, health information delivered was not based on any guidelines but rather was based on content adolescents wished to receive. Dialogflow software was used to develop the intervention, and behavior change was promoted using the Health Action Process Approach model [36]. Moreover, there was 1 (20.0%) study that examined a virtual reality–based mobile game that was supported by AI (food robot), which was different from the other 4 (80.0%) chatbots and used personalized behavior reinforcement to increase awareness and self-efficacy [33]. Only 2 (40.0%) of 5 studies used any codesign with the end user, and no studies involved parents or caregivers in the intervention development.

### Youth Consultation

The youth consultation led to the statement seen in Textbox 1. In brief, adolescents had concerns around (1) information the chatbots delivered being misleading or harmful and (2) ethical concerns around the privacy of data collected and misunderstanding of individual circumstances that may provide inaccurate health advice.

**Youth statement in their own words.**

Chatbots have great potential in the field of health promotion, particularly in areas that encompass physical activity and nutrition. However, there are many factors that must be considered before they are implemented in such a field. The extensive growth and use of social media and the sharing of public information [have] seen society enter a world of fake, or rather, misleading information. This has created an environment where it is hard to navigate what is the truth and what is harmful. Therefore, any information that the chatbots release must be highly regulated and fact-checked before [being] released. So many misleading and often harmful nutritional messages are put out to audiences that [result] in body dysmorphia, decreases in self-esteem, and eating disorders. The information used must be phrased in a manner that is not triggering nor encouraging such poor habits. To increase their acceptance in the wider population, the chatbot should be associated with a brand or source that already has a “trusted” label. This would make audiences more likely to engage with it.

The ethical concerns of chatbots for uses in health promotion can be divided into 2 main categories: the potential for chatbots to exploit young people for commercial gain and the potential for chatbots to cause harm to young people through the provision of inaccurate health advice. There are several ways in which chatbots could exploit young people for commercial gain. Chatbots could be used to sell young people’s personal data to third parties or to generate targeted advertising based on young peoples’ health conditions. Chatbots could also be used to upsell young people on expensive treatments, exercise programsl, or supplements. To minimize the risk of chatbots exploiting young people for commercial gain, it is important to ensure that chatbots are transparent about how they will use any personal data that they collect. Young people should also be given the option to opt out of any data collection or advertising. There is also a risk that chatbots could cause harm to young people through the provision of inaccurate health advice. This could happen if chatbots are not based on credible health sources or if they are not able to properly understand young people’s individual circumstances. To minimize the risk of chatbots causing harm to young people, it is important to ensure that chatbots are only used as a supplement to, and not a replacement for, health advice from a qualified health care professional.

## Discussion

### Principal Findings

This systematic scoping review evaluating chatbots in promoting nutrition and physical activity behaviors in adolescent populations is an emerging and underresearched field. The 5 published studies found insufficient evidence for the acceptability and feasibility of chatbots. Only 2 of the 5 included studies found adolescents were satisfied with the chatbot used in the intervention [32,33]. The chatbots demonstrated modest efficacy in improving adolescents’ nutrition, physical activity behaviors, and knowledge. The chatbots were used within mobile apps or mobile services with differing design features, including conversational agents, gamification, personalized feedback, and monitoring of behavior change. Adolescents from the youth advisory group presented unique insights into the use of chatbots in nutrition and physical activity interventions, including ethical concerns and the use of false or misleading information, which was not otherwise identified in the published literature. Taking these findings together, this review found that there is limited evidence for the feasibility and acceptability of chatbots in promoting nutrition and physical activity behaviors. Therefore, together with our youth advisory group, we propose suggestions for improved chatbot development and research study design.

### Comparison With Existing Literature

To the best of our knowledge, this is the first systematic scoping review of chatbots in promoting nutrition and physical activity behaviors in adolescent populations. Chatbots have been broadly used in chronic disease prevention and management. A systematic review conducted by Laranjo et al [37] demonstrated that conversational agents are most commonly used in mental health management, resulting in reduced depression symptoms, improved narrative skills scores in autism, and suicide prevention [37]. This review also highlighted conversational agents (1) supporting patients with type 2 diabetes for physical activity and diet behavior change and self-management practice and (2) supporting clinicians and hypertension patients in telemonitoring and data collection. In this review, 12 of the 14 studies reported user experience. Dissimilar to our findings, most reported high overall satisfaction. Of the 2 studies that included adolescent participants, the chatbot designed for self-management of a specific condition (asthma) [38] had a higher overall satisfaction compared to the chatbot designed for education (sexual health and substance abuse) [39]. In our review, participants reported greater satisfaction with chatbots for self-monitoring of food consumption and dietary intake. However, most participants reported that chatbots were often not easy to use and sometimes forgot to record their dietary intake [32]. Consequently, participants tended to underreport their dietary intake using the app in comparison to other validated dietary recall methods. This may explain why studies in our review, which were focused on prevention and risk factor modification, not chronic disease self-management, had lower overall satisfaction. This also demonstrates the need to focus on design features.

There are other studies focusing on the feasibility of chatbots used in adolescents but not limited to nutrition and physical activity behavior change. A chatbot called Tess (X2 AI) using AI was found to be an engaging and feasible approach to support weight management and counseling in adolescents and children [40]. Participants reported Tess to be useful 96.0% of the time. The high level of satisfaction compared to the studies included in our review may be explained by the different length of conversations participants can have with Tess. Tess can offer large amounts of message exchanges, which demonstrates high engagement, attraction, and acceptability of AI chatbots [40]. It should be noted that Tess is a commercially available service with a customizable platform where the content can be tailored for specific populations or interventions. This is unlike the chatbots evaluated in this review, which were developed by the research teams for the purpose of 1 intervention. Consequently, integrating language techniques may be useful to incorporate into the chatbot database to enhance engagement with adolescents and stimulate longer message conversations, covering topics outside of the intervention itself.

NLP may be a good choice for chatbot database design for adolescents if databases can be developed to offer small talk and noninterventional questions, in addition to the intervention. In Maenhout et al’s [36] study, adolescents found it frustrating if the chatbot misunderstood their question. A conservational agent that uses NLP may make them feel like they are communicating with another human, which in turn may enhance engagement and the user experience [36]. In a similar study, a chatbot using NLP that was focused on improving physical activity in adults found the chatbot increased participants’ step count and self-reported physical activity. Most participants scored the chatbot as OK (78.8%), and one-third of the participants were interested in continuing using the chatbot following the study [41]. For NLP to be successful, it is vital to engage adolescents throughout the database design process to develop the database with youth-oriented language and enhance the feeling of communicating with another human. A scoping review by Kramer et al [42] found that conversational agents for coaching people in a healthy lifestyle were often designed for the end user rather than with the end user. In this review, only 2 of the 5 chatbots incorporated any kind of codesign with adolescents in the development of the chatbot intervention, which may explain the low satisfaction with the chatbots as they were not designed with the end user, and therefore do not meet the adolescents’ needs.

The youth consultation uncovered insights into the use of chatbots for nutrition and physical activity interventions that were not identified in the published literature. One of the suggestions raised by adolescents was to have the chatbot associated with a brand. In a previous study, adolescents identified that the most helpful lifestyle health information online comes from a credible and reliable source [43]. Adolescents are highly brand conscious [44], and therefore, having the chatbot associated with a brand may increase their trust in the information that is being presented to them. Another insight raised by adolescents was around the provision of inaccurate health advice that may cause harm. To counter this, appropriate monitoring of chatbot conversation logs is vital in future studies to ensure chatbots do not deviate and provide incorrect information to adolescents. Conversation logs must also be monitored to ensure any self-disclosure from the adolescent to the chatbot is communicated and actioned accordingly. In the studies included in this review, there was no potential for chatbots to provide incorrect information as none of the conversational agents used AI to provide responses. Only 1 of the studies in this review applied monitoring of conversation logs, yet it was to assess the feasibility and not for safety. For chatbots to be both safe and effective in the future, researchers and developers must work together to obtain information about adolescents and their individual situation and then tailor accurate health information that is best suited to their needs. Furthermore, safeguards need to be in place to ensure the safety of adolescents while using chatbots for health promotion interventions [45], especially if future chatbots are developed using AI. Rigorous beta-testing of the intervention should occur before being implemented to ensure that interventions are relevant, appealing, functional, stable, and useful [46]. In addition, exposure time to the chatbots must also be considered in future interventions to ensure that adolescents do not increase their screen time beyond the recommended guidelines.

### Limitations

This scoping review demonstrates the limited published literature on chatbots used in the adolescent population for nutrition and physical activity behavior promotion. It must be noted that there are some limitations to this research. First, not all studies provided data on the feasibility and acceptability of the chatbots, which is crucial to understanding barriers and enablers to implementing such an intervention on a wider scale. Second, none of the studies included in this review that included a conversational agent used AI. Chatbots based on AI are trained to respond to queries based on texts to which they are exposed; therefore, the training of AI chatbots could not be assessed within the scope of this review. Next, we only included peer-reviewed published studies. There is the potential of other studies that would otherwise fit the criteria of this review. Finally, youth consultation is a strength of our review; however, it was conducted in a group of Australian adolescents, so the results may not be generalizable to other populations.

### Conclusion

Limited research is available on the use of chatbots in adolescent nutrition and physical activity interventions, finding insufficient evidence for the acceptability and feasibility of chatbots in the adolescent population and only minor improvements in health-related outcomes due to the interventions. Similarly, adolescent consultation identified important issues relating to the design features that were not mentioned in the published literature. Researchers and developers should consider codesigning chatbots with adolescents to ensure that they are feasible and acceptable to an adolescent population.

## Appendix

Multimedia Appendix 1 Search strategy.

Multimedia Appendix 2 Characteristic of chatbots and interventions.

## Abbreviations

AI: artificial intelligence
HAPYUS: Health Advisory Panel for Youth at the University of Sydney
NLP: natural language processing
PBA: person-based approach
PRISMA-ScR: Preferred Reporting Items for Systematic Reviews and Meta-Analyses extension for scoping reviews
RCT: randomized controlled trial
WHO: World Health Organization
