# Supplementary material for: Feasibility and Acceptability of Chatbots for Nutrition and Physical Activity Health Promotion Among Adolescents: Systematic Scoping Review With Adolescent Consultation
Source: JMIR Hum Factors. 2023 May 5;10:e43227. doi: 10.2196/43227 (PMC10199392; doi:10.2196/43227)
Supplement: Multimedia Appendix 1 [file humanfactors_v10i1e43227_app1.docx]

# Multimedia Appendix 1: Search Strategy (Ovid MEDLINE(R) ALL <1946 to March 18, 2022>)

1. chatbot*.mp.
2. chatterbot*.mp.
3. artificial intelligence/ or machine learning/
4. (artificial* intelligen* or machine learning).mp. (
5. ((Conversation* or chat* or live chat or virtual) adj3 (agent* or AI or Artificial* intelligen* or interface* or software or textual or program* or application* or app or apps or digital or technolog* or assistant* or bot* or system*)).mp.
6. voice technolog*.mp.
7. voice assistan*.mp.
8. (assistan* adj2 (google or virtual or intelligent)).mp.
9. (apple adj2 siri).mp.
10. amazon alexa.mp.
11. (Text messag* or SMS or Short messag* service* or Dialogue or Conversation* or Communication).tw.
12. Smart Wireless Interactive Healthcare System.mp.
13. Ehealth.mp. or Telemedicine/
14. Digital assistant*.mp.
15. dialog* system*.mp.
16. telemedicine*.mp.
17. (Mobile health or Mhealth or Electronic health or Ehealth).tw.
18. 1 or 2 or 3 or 4 or 5 or 6 or 7 or 8 or 9 or 10 or 11 or 12 or 13 or 14 or 15 or 16 or 17
19. nutrition therapy/ or diet therapy/
20. nutrition therap*.mp.
21. diet therap*.mp.
22. exp Diet/
23. exp Food/
24. exp Healthy Lifestyle/
25. ((diet* or nutrition*) adj3 (intervention* or program* or education* or counsel* or advis* or therap* or behavio?)).mp.
26. healthy eat*.mp.
27. ((diet* or nutrition* or energy) adj2 intake*).mp.
28. 19 or 20 or 21 or 22 or 23 or 24 or 25 or 26 or 27
29. exp Exercise/
30. exercis*.mp.
31. physical activit*.mp.
32. exp Physical Fitness/
33. physical fitness*.mp.
34. ((exercise* or physical activit*) adj3 (intervention* or program* or education* or counsel* or advis* or therap* or behavio?)).mp.
35. 29 or 30 or 31 or 32 or 33 or 34

36 18 AND 28

37 18 AND 35

38 36 OR 37

39 - LIMITS - human, 2010-current, All child AND adolescent
