# Supplementary material for: Feasibility and Acceptability of Chatbots for Nutrition and Physical Activity Health Promotion Among Adolescents: Systematic Scoping Review With Adolescent Consultation
Source: JMIR Hum Factors. 2023 May 5;10:e43227. doi: 10.2196/43227 (PMC10199392; doi:10.2196/43227)
Supplement: Multimedia Appendix 2 [file humanfactors_v10i1e43227_app2.docx]

**Multimedia Appendix 2. Characteristic of chatbots and interventions.**

| First Author, Year, Country | Type of chatbot | Details of chatbot | Targeted behaviour | Details of intervention | Length of intervention | Follow-up(s) | Primary outcomes | Secondary outcomes | Key Findings |  |
| --- | --- | --- | --- | --- | --- | --- | --- | --- | --- | --- |
| Lee, 2017, Korea (16) | Mobile application, “Diet-A” | A dietary self-monitoring application that allows records keeping of dietary intake, real-time feedback, and provision of information on disease prevention. | Nutrition | Participants used the Diet-A application to record their dietary intake (foods, dishes, and beverages) | 3 months | 3 months | A feasibility questionnaire adapted from previous studies was used post intervention. A five-point Likert scale measured the degree of satisfaction, convenience, and efficiency. | The CAN-Pro 4.0 program was used to assess nutrient intake. Data was obtained from one-day 24-hr recalls and dietary habit questionnaires pre and post intervention. | Significant decrease in sodium and calcium intake.  62% of participants were satisfied using the application. Nutrient intake estimates were lower in Diet-A compared to 24-hr recall.  71% found it was burdensome to record their diet. |  |
| Padman, 2017, India (17) | Artificial Intelligence “Fooya!” | A virtual reality based immersive mobile game to achieve personalized behaviour reinforcement | Nutrition | Participants played the mobile game for up to 20 minutes, making food choice decisions, and destroying unhealthy food robots to save themselves. | 2 months | N/A, retrospective analysis | Pre and post-test  questionnaire collected demographic data, food habits, self-efficacy in selecting healthy food habits, frequency, and use of playing video games. Gameplay measurement variables from app clickstream data use (for e.g., Type of nourishing food “consumed”). | Gameplay measurement variables from app clickstream data (for e.g., Total gameplay time, measuring per level and overall game engagement) | Children had increased awareness of healthy vs. unhealthy foods.  Children are interested in using different game mechanics built into games. There was decreased engagement and motivation to look up nutritional facts as participants progressed in the game. Further design methods are needed to retain interest. |  |
| Pyky, 2017,  Finland (18) | Mobile service “MOPOrtal” | Web-based interface where participants received tailored, automated health information and feedback messages based on their goals; physical activity instructions and guidelines; mixed-reality conquering game, with social- networking possibilities. | Physical activity | Participants wore the activity monitor daily and uploaded their activity data. They received tailored feedback through the MOPOrtal. | 6 months | 6 months  Monthly rating of stage of exercise behaviour change (transtheoretical model) | Daily minutes of physical activity:  Wrist worn monitors (Polar Active, Polar Electro Ltd., Finland), self-reported questionnaire  BMI: wall mounted tape and weight scale (InBody720, Biospace Co Ltd). | Self-reported questionnaire; life satisfaction (four-item  scale measuring happiness, interest in life, feelings of loneliness  and ease of living), and self-rated health | Limited increase in physical activity and increased mean weight in both groups.  Improved life satisfaction occurred post intervention regardless of group allocation.  Men with low life satisfaction and poor self-rated health at baseline was associated with improved satisfaction post intervention. | |
| Stasinaki, 2021, Switzerland (19) | Mobile application “PathMate2” | Two chat channels: (1) a conversational agent (virtual coach) chat with and encourage participants. (2) HCP (human coach) can chat with participants. | Physical activity | Participants were given a smartphone with the PathMate2 app involving daily interaction with the conversational agent to influence physical activity. A self-responsible maintenance phase followed to monitor behaviour change. | 12 months with two phases: 5.5-month intensive intervention phase (T1), 6-month maintenance phase (T2) | 5.5 months, 9 months, 12 months. | BMI-SDS: adjusted  according to the LMS method for sex, age as well  as the skewness of BMI distribution, (WHO standard) | Changes in body fat and muscle mass (bioelectrical impedance analysis), waist-to-height ratio, physical capacities (modified Dordel-Koch-Test), blood pressure and pulse.  Biofeedback relaxation exercises were measured using plasma cortisol and stress questionnaires | The app combined with standardized counselling  achieved significant improvements of body fat and physical capacities, i.e., increased muscle mass during the study. However, there was no sustained significant change of BMI-SDS in both groups. | |
| Maenhout, 2021, Belgium | A self-regulation mobile app and chatbot | Adolescents engaged in conversation with the chatbot, asking questions about health promoting behaviours. Answers were drawn from a updated database following phase 2 conservation logs. | Physical activity and nutrition | An adapted chatbot protype was tested in a pilot study using a person-based approach. Participants could ask the chatbot questions about physical activity, sedentary behaviour, breakfast intake, and mental health. | 2 weeks (phase 3) | 2 weeks | Phase 3:  Process evaluation interviews using a  semi-structured interview guide based on the Medical Research Council Framework.  Engagement questionnaire using  Digital Behaviour Change Intervention (DBCI) Engagement Scale and the User Engagement Scale (UES)  Qualitative analysis of conservation logs with the chatbot | Phase 1: Focus groups coded and analysed using NVivo 12 software  Phase 2:  Qualitative analysis of conservation logs with the chatbot. | Engaging adolescents throughout each development phase led to greater insights about user preferences. These included: confidentiality; youth centred language and culture; and personalisation.  Users wanted a greater database for chatbots to engage in small talk and non- intervention related questions.  Despite extensive co-design, user engagement was moderate. | |
